# The hidden role of buffalo trade network in bovine epidemic spreading

**DOI:** 10.1371/journal.pone.0313657

**Published:** 2024-11-13

**Authors:** Giacomo Zoppi, Luca Candeloro, Lara Savini, Vittoria Colizza, Mario Giacobini

**Affiliations:** 1 Data Analysis and Modeling Unit, Department of Veterinary Sciences, University of Torino, Grugliasco, Italy; 2 Istituto Zooprofilattico Sperimentale dell’Abruzzo e del Molise *G. Caporale*, Teramo, Italy; 3 INSERM, Sorbonne Université, Pierre Louis Institute of Epidemiology and Public Health, Paris, France; University of Messina: Universita degli Studi di Messina, ITALY

## Abstract

Animal movements are a key factor in the spread of pathogens. Consequently, network analysis of animal movements is a well-developed and well-studied field. The relationships between animals facilitate the diffusion of infectious agents and, in particular, shared environments and close interactions can facilitate cross-species transmission. Cattle are often the focus of these studies since they are among the most widely distributed and traded species globally. This remains true for Italy as well, but with an important additional consideration. Indeed, another important productive reality in the peninsula is buffalo farming. These farms have an interesting characteristic: approximately two-thirds of them also rear cattle. This coexistence between cattle and buffalo could have an impact on the diffusion of pathogens. Given that buffalo farms are often overlooked in the literature, the primary goal of this work is to investigate the potential consequences of omitting buffalo from cattle network analyses. To investigate this impact, we will focus on Q fever, a disease that can infect both species and is present on the Italian territory and for which the impact of the buffalo population has not been thoroughly studied, and simulate its spread to the farms of both species through compartmental models. Our analysis reveals that despite the significant difference in network sizes, the unique characteristic of Italian buffalo farms makes the buffalo network essential for a comprehensive understanding of bovine disease dynamics in Italy.

## Introduction

### Network modeling of animal diseases

Network analysis of animal movements is one of the most developed fields that studies and tries to understand the dynamics of disease diffusion. Animals, both wild and domestic, play a key role as they can act as reservoirs, vectors, or hosts for various pathogens. Modeling those interactions as complex networks allows us to obtain a comprehensive understanding of how the diseases spread within or between different populations. In addition to this, it can help to develop strategies for disease control or prevention [1–6].

So, animal networks play a crucial role in the diffusion of diseases as they create pathways for the spread of pathogens between species. The interconnected relationships among animals facilitate the transmission of infectious agents, with the movement of infected individuals contributing to the geographical dissemination of diseases. Cross-species transmission is facilitated by shared environments and close interactions, making animal networks key contributors to the emergence and persistence of diseases. Their role is indeed crucial, especially given the impact various pathogens can have on both animals and humans. Several pathogens have been studied with this approach.

In the context of brucellosis, a contagious zoonotic disease caused by *Brucella* bacteria, domestic animals such as cattle, sheep, and goats can spread the bacteria, leading to the contamination of their fluids, like milk or reproductive discharges. This poses a risk not only to other animals within the same herd but also to humans who come into contact with infected animals or consume untreated dairy products. Additionally, bovine brucellosis harms the farm economy, due to decreased fertility, increased abortion rates, and reduced milk and meat production [7–9].

Tuberculosis, caused by *Mycobacterium tuberculosis*, is another example where animal networks play a significant role. While tuberculosis primarily affects humans, it can also infect a variety of animals, including cattle (bovine tuberculosis) and wildlife. Cattle are the primary hosts, but the disease can spread among various animals, including buffaloes and wildlife. Close contact between humans and animals in certain regions facilitates disease transmission between species [10–14].

Bovine Viral Diarrhea (BVD) is a widespread viral infection affecting cattle worldwide. It is caused by the Bovine Viral Diarrhea Virus (BVDV) and the virus primarily targets the gastrointestinal and respiratory systems of cattle, leading to symptoms such as diarrhea, respiratory distress, reduced milk production, and reproductive disorders. BVDV poses a significant economic threat to the livestock industry due to its ability to cause persistent infections, immunosuppression, and reproductive failures while being difficult to diagnose [15–17].

Bovine Infectious Rhinotracheitis (IBR) is a highly contagious viral disease affecting cattle. It is caused by bovine herpesvirus type 1 (BoHV-1) and primarily manifests as upper respiratory tract infections. It is caused by an alphaherpesvirus, thus establishing a lifelong latent infection. The latent virus can be reactivated by stressful stimuli, including environmental heat, transporting cattle long distances, and restriction of water and food [18]. IBR can have significant economic consequences for the livestock industry, as it can compromise the health and well-being of cattle, leading to reduced milk production, growth rates, and meat quality [19–21].

Q fever is an infectious disease caused by the bacterium *Coxiella burnetii* [22–24]. This pathogen can infect a wide range of animals, including mammals (also humans), birds, reptiles, and others. In animals, the disease usually does not cause visible symptoms but, when it manifests clinically, it can induce abortions or infertility and so can generate a significant economic impact for affected farms [25, 26]. Infected ruminants excrete *Coxiella burnetii* through different biological fluids including milk, feces, urine, as well as birth or abortion products [27–29] and it can survive several weeks in nature (eg. in contaminated hay) and be spread by the wind. Animals can be infected through the fluids just mentioned, by inhaling the bacteria, or through tick bites.

Cattle, sheep, and goats are considered among the primary reservoirs for human contamination [22, 30, 31]. Aerosol is the primary mode of human contamination, it may occur directly from fluids of infected animals and human-to-human transmission is extremely rare, although there have been cases of this through blood transfusions [32]. Many infected people have no symptoms but it can cause fever, headache, diarrhea, and vomiting. In some severe cases, it can result in pneumonia or hepatitis. Women infected during pregnancy may risk miscarriage or preterm delivery [27]. A small percentage of people (≤ 5%) develop a more serious infection known as chronic Q fever. This can lead to endocarditis and may be fatal if not treated properly. In 2021, there was an outbreak of 14 cases among tourists in Italy caused by an infected calf. This incident highlighted the problem of animal-to-human transmission and the challenges of diagnosing the disease [33]. Due to its zoonotic effects and economic implications for livestock [25], Q fever represents a significant concern that impacts both public and animal health.

In the above-mentioned studies, usually, the disease is treated considering only one species. For example, cattle are often mentioned because they are among the most widely distributed and traded species globally. Narrowing the analysis to Italy, this fact remains true. However, some peculiarities might be important to consider.

### Potential role of buffalo farms

Buffalo farms constitute a significant productive reality in the peninsula and possess an interesting characteristic: approximately two-thirds of these farms also rear cattle. Henceforth, we will refer to these farms as *mixed* farms.

**Fig 1:**
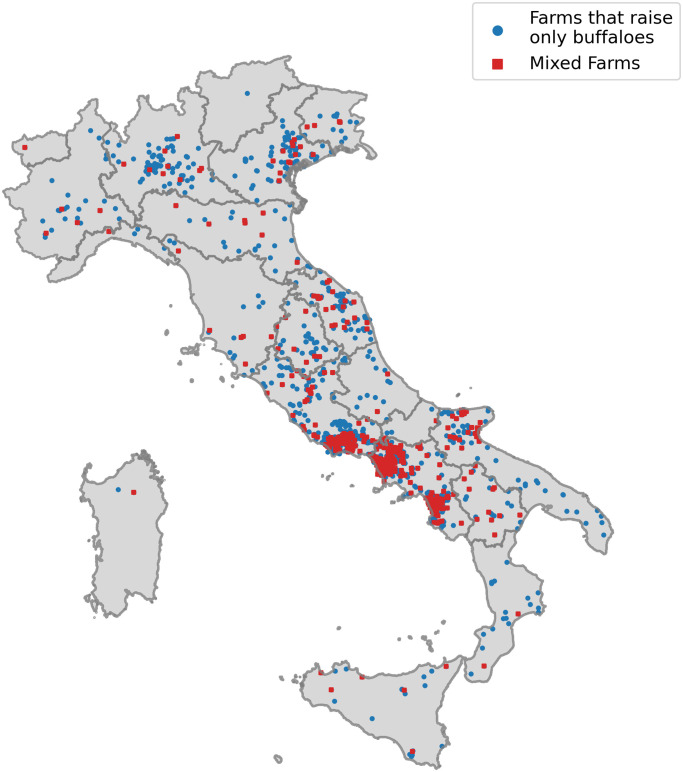
Geographical distribution of buffalo farms in Italy. Blue dots: Farms that raise only buffaloes. Red squares: *mixed* farms. Shapefile of Italy from GADM data under a CC-BY license https://gadm.org/license.html.

Fig 1 illustrates with blue dots the geographical distribution of the farms exclusively housing buffaloes, and with red squares the distribution of *mixed* farms in Italy. It is possible to see how the disposition of the farms is similar, with a higher density of those in the southern region of the Italian peninsula, therefore excluding a possible regional-restricted presence of *mixed* farms.

This coexistence of cattle and buffalo within the same environment raises questions about the potential impact it may have on the transmission and survival of different pathogens between these two species. Hence, the often overlooked movements associated with the buffalo industry may significantly contribute to the dissemination of pathogens.

Given these considerations, the primary objective of this study is to understand the impact resulting from the omission of the presence of buffaloes, leading to potential inaccuracies in the evaluation of the overall situation.

In recent times, studies on the prevalence of *Coxiella burnetii* among buffaloes in Italy [26, 34, 35] have found a wide distribution of this pathogen. Thus, considering the particular situation of *mixed* farms and the presence of this pathogen in both cattle and buffalo, a relevant role of the latter can be assumed. Therefore, not considering the totality of movements could lead to underestimating the prevalence of this pathogen or to making incorrect or inefficient decisions.

Q fever is a disease that can infect both species and is present on the Italian territory and for which the impact of the buffalo population has not been thoroughly studied. If their role is confirmed through this analysis, one should contemplate incorporating the buffalo network into network models, particularly those focused on bovine diseases in Italy.

## Methods

To analyse the role that the buffalo population could have in the spreading of this disease, we considered the movements related to the trades of both cattle and buffaloes in Italy. We extracted data from the Italian National Animal Identification and Registration Database (NDB) of the Ministry of Health, obtaining the starting point, ending point, and date of each movement. We restricted our analysis to national movements for 4 years, from 2017 to 2020, the period in which the data are more stable and solid. We also removed movements to slaughterhouses because of no epidemiological interest.

### Dataset description

For each movement, along with the date, we have information on the type of departure and arrival points, as well as their geographic locations. Our analysis will focus on the farms, categorized by the species they contain. We will examine the number of movements related to each species, taking a network approach to analyse the number of movements arriving at (indegree) or starting from (outdegree) a farm.

**Fig 2:**
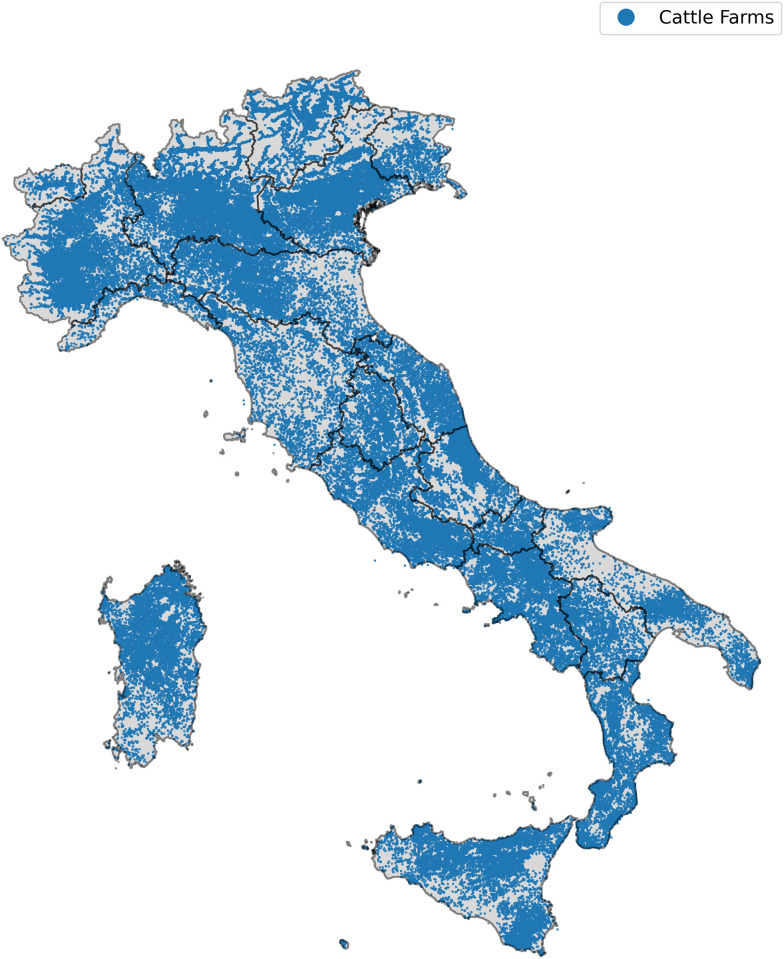
Geographical distribution of cattle farms in Italy. Shapefile of Italy from GADM data under a CC-BY license https://gadm.org/license.html.

The following definitions will be used:

- Cattle farms: all the farms that house cattle, visible in Fig 2.
- Cattle network: a network that considers all the movements between different entities housing cattle (i.e., cattle farms and mixed farms).
- Buffalo farms: all the farms that house buffaloes.
- Buffalo network: a network that considers all the movements between different entities housing buffaloes (i.e., buffalo farms and mixed farms).
- *Mixed* farms: farms that house both buffaloes and cattle.

Table 1 considers the cattle network, so it displays the aggregate count of movements associated with either the origin or destination being a cattle farm, along with the count of *active* cattle farms. *Active* cattle farms are those farms that are either the origin or destination for at least one movement in a given time span. The data is grouped by year, revealing a gradual decline in both the overall number of movements and the count of active cattle farms throughout the years.

**Table 1:** Cattle network. Number of active farms and movements by year.

year	active cattle farms	movements between cattle farms
2017	99641	667315
2018	95657	654527
2019	93107	650713
2020	92491	642949

Table 2 displays the same data as the previous one but considers the buffalo network. Here, “active *mixed* farms” are those *mixed* farms that are either the origin or destination for at least one movement of buffaloes in a given period.

**Table 2:** Buffalo network. Number of active farms and movements by year.

year	active buffalo farms	movements between buffalo farms	active *mixed* farms	movements between *mixed farms*
2017	1243	4791	853	4390
2018	1242	5327	850	4705
2019	1237	6372	839	5352
2020	1305	8075	870	6609

Here can be seen how the Italian reality of this species has a different order of magnitude than that of cattle. This adds significance to our research question. While the size of this network might suggest it is negligible, the seroprevalence of Q fever, as discussed in the introduction, appears to conflict with this idea.

It is also interesting to note how the number of movements is significantly increasing despite a small rise in the number of active farms, suggesting a growing average number of movements per farm.

In 2017, movements to or from buffalo *mixed* farms accounted for more than 90% of the total buffalo movements. By 2020, this percentage had decreased to 80%.

One possible explanation for this shift is that in the past, companies with existing cattle facilities could easily expand to include buffaloes without a significant initial investment. As the market for buffalo milk products, such as buffalo mozzarella, grew in Italy [36], there was a trend toward establishing farms exclusively dedicated to buffaloes.

In Table 3 we have a brief statistical analysis of the yearly aggregated, directed, and with possible multiple edges between the same nodes network [37]. The weight of the edges is the number of animals moved in a single movement. In this table, *mixed* farms (buffalo network) denotes the subset of *mixed* farms that are either the origin or destination for at least one movement of buffaloes in a given period. The mean degrees are computed on nodes that have that degree different from 0.

**Table 3:** Statistics of yearly aggregated networks by species.

year	Nodes	Mean indegree	Mean outdegree	Mean weight	Density
2017	cattle farms	8.32	8.83	54.73	5.64e-05
	buffalo farms	5.86	5.78	38.48	0.0029
	*mixed* farms (buffalo network)	6.11	6.01	37.78	0.0032
2018	cattle farms	8.37	8.82	56.43	5.90e-05
	buffalo farms	6.43	6.14	41.09	0.0031
	*mixed* farms (buffalo network)	6.66	6.27	40.39	0.0033
2019	cattle farms	8.53	8.91	58.05	6.16e-05
	buffalo farms	8.65	6.97	45.99	0.0038
	*mixed* farms (buffalo network)	9.03	7.06	44.59	0.0040
2020	cattle farms	8.35	9.04	59.81	6.17e-05
	buffalo farms	10.61	8.39	49.09	0.0044
	*mixed* farms (buffalo network)	11.04	8.62	48.21	0.0048

Also from this data, we can see the different sizes of the networks of the two species and the increasing trend related to the buffalo farms.

### Disease model

We will then simulate the spread of Q fever, using a compartmental model in discrete time as it allows us to maintain the movement generated network structure and it is widely used in literature [38, 39]. In this model, we consider the farms as the epidemiological units, since the diagnosis of Q fever is often done at the farm level. Therefore, we employ an SIS model, which accurately reflects the dynamics at this scale. More complex models, incorporating additional compartments, are better suited for capturing individual animal-level processes. The model divides the farms into two compartments: susceptible (S) and infectious (I). A susceptible farm is a farm where no animals are infected with the disease. An infectious farm is a farm where at least one animal is infected with the disease. The model assumes that the disease can spread from an infectious farm to a susceptible farm through the movement of animals. So, a susceptible farm that receives animals from an infectious farm will also become infectious according to a defined probability of infection. An infectious farm becomes susceptible again, according to a defined probability of healing, when all infected animals are removed or healed, and so there are no animals infected with the disease on the farm. Finally, given the difficulty in accurately estimating infection and recovery rates at the farm level, we conducted a grid search to explore a range of parameter values in our simulation analysis.

## Network analysis

From the extracted data we built weekly temporal networks where the premises of origin and destination will be the nodes and the movements of the animals will be the edges. In this network, the edges have a direction (from the origin to the destination) and a weight given by the number of animals moved [40]. We aggregated the movement data by week and created a series of networks to capture temporal characteristics that would be lost with a static approach [1, 41–43].

### Cross-species interaction

**Fig 3:**
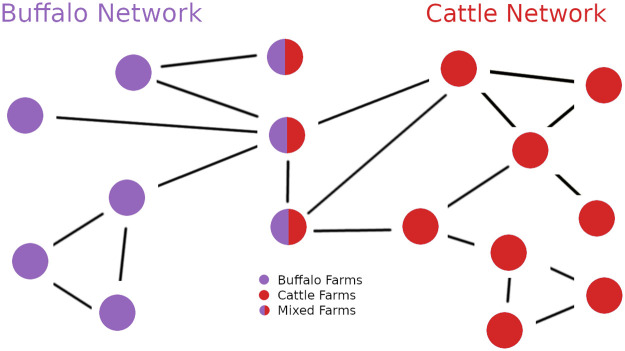
Cattle and buffalo networks interaction.

As previously mentioned, the coexistence of cattle and buffaloes in *mixed* farms establishes a potential link between the buffalo and cattle networks, providing a pathway for the transmission of diseases between the two. Nodes, where both species are present, can help the spread of diseases, facilitated by airborne transmission or through vectors (eg. ticks). In Fig 3 we have a depiction of this concept. To model this aspect in our disease model and enable the potential transmission of infection between the buffalo and cattle networks, we created links between those two through the *mixed* farms [44].

### Cattle network

**Fig 4:**
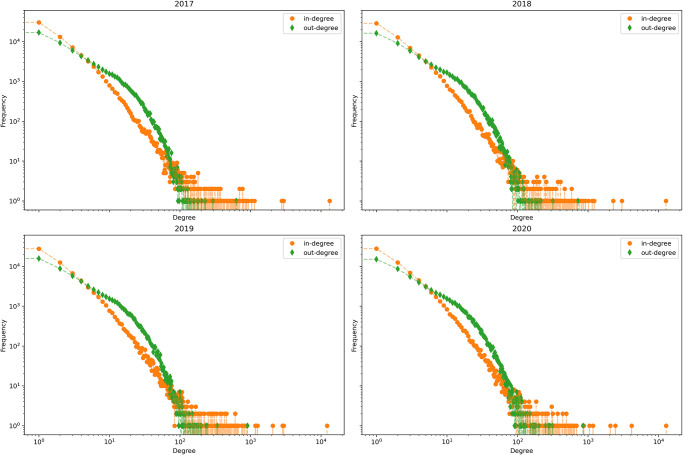
Degree distribution—Cattle network, yearly aggregation.

Fig 4 presents the aggregated indegree and outdegree distributions of the cattle network for each year. The four plots show similar, highly heterogeneous patterns. Indeed, we can see how we have some farms with an high indegree while the same is not true regarding the outdegree. This disparity might indicate a network structure where larger farms buy from different smaller ones.

**Fig 5:**
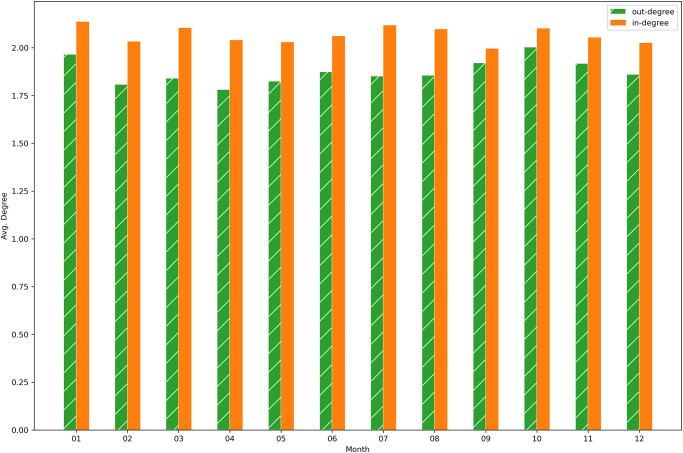
Average degree per month—Cattle network. Monthly average across all active nodes for each of the four years.

This shows also in Fig 5, where the indegree and outdegree for each month, averaged on the four years, are computed. Consistent with the previous observation, also the average indegree remains slightly higher throughout the months.

**Fig 6:**
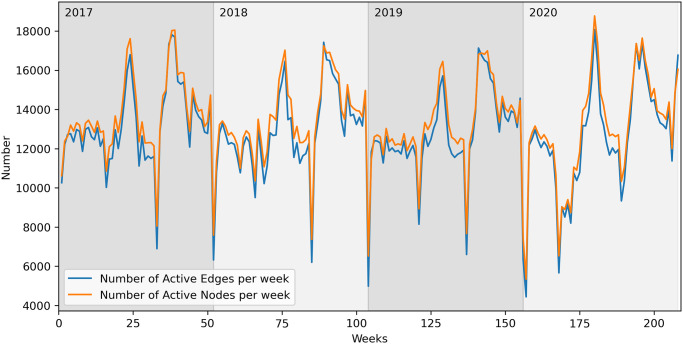
Active nodes and edges—Cattle network, weekly aggregation.

In Fig 6, the number of active edges and active nodes per week are plotted. Several oscillations occur, caused by seasonal movements typical of the Italian scenario, such as summer grazing in pastures [1, 7, 40]. Despite the fluctuating values, the general behaviour seems to be constant. Additionally, we observe that the number of active edges is similar to the number of active nodes, suggesting that while there is a significant number of active farms per week, they do not engage in a large volume of movements.

**Fig 7:**
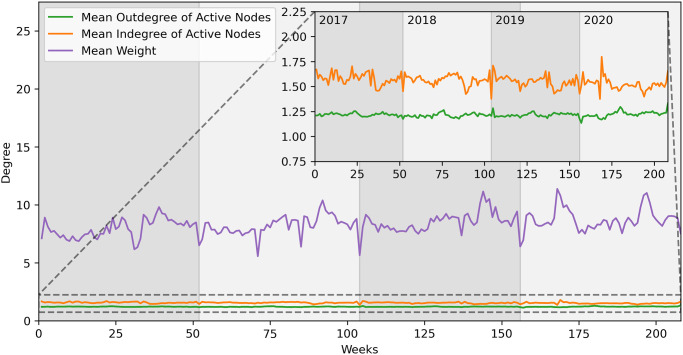
Cattle network statistics—Weekly aggregation. Main plot: mean outdegree, indegree and weight. Secondary plot: magnification of outdegree and indegree.

In Fig 7, this characteristic is evident. Here, we have plotted, for each week, the mean indegree and outdegree computed among the active nodes, along with the mean number of animals in each movement (weight). The mean degrees are computed on nodes that have that degree different from 0. On the main plot, we see that the average number of animals moved is relatively low. Additionally, on the zoomed plot, we observe how the indegree and outdegree attain modest values, never exceeding 2. The indegree is higher than the outdegree, suggesting that smaller farms are moving towards the same node.

That being said, the number of active farms is significantly large, as evident in Fig 2, where we highlighted the geographical location of all the active farms over the 4 years. These nodes also exhibit strong connectivity. Indeed, the largest weakly connected component, considering only movements from farm to farm each year, encompasses 80% of the farms (and more than 95% if we consider also the movements from and towards the pastures). The largest strongly connected component, instead, is composed by almost 50% of the nodes. This, along with the total number of movements, needs to be taken into account when analysing the potential implications for the diffusion of a disease.

### Buffalo network

**Fig 8:**
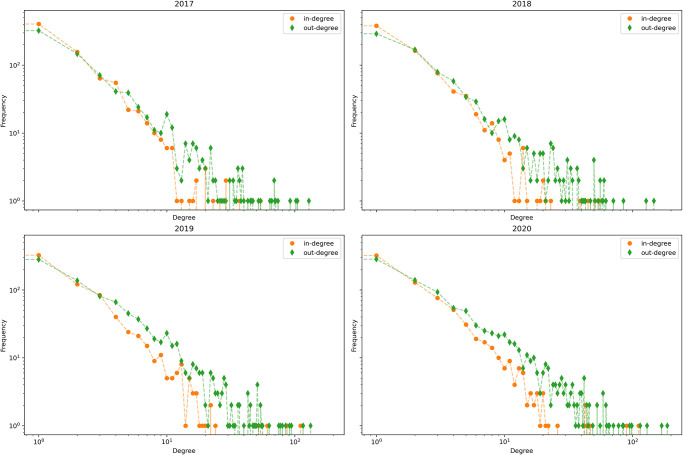
Degree distribution—Buffalo network, yearly aggregation.

The indegree and outdegree distributions for the buffalo network are also heterogeneous, as is visible in Fig 8. In this figure, we can find some farms that have a high outdegree. It should be noted that the values here are much smaller than those related to the bovine network.

**Fig 9:**
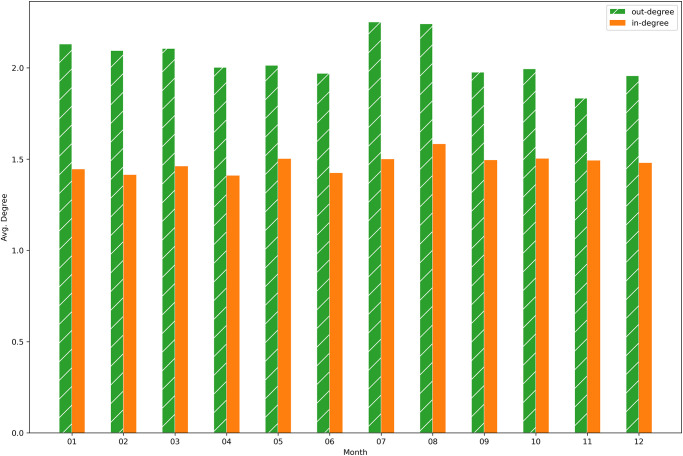
Average degree per month—Buffalo network. Monthly average across all active nodes for each of the four years.

Fig 9, where the indegree and outdegree for each month, averaged on the four years, are computed, is consistent with the previous figure. Indeed, also the average outdegree is slightly bigger than the average indegree for each month.

**Fig 10:**
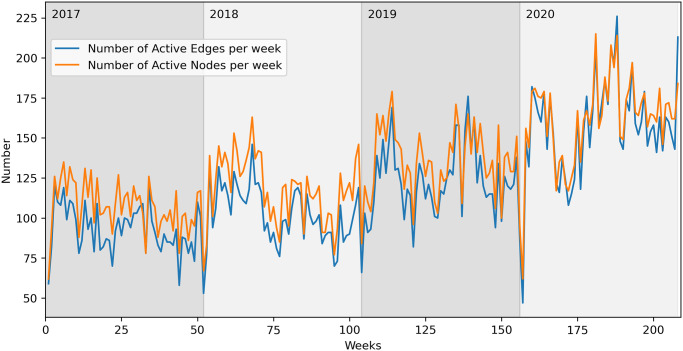
Active nodes and edges—Buffalo network, weekly aggregation.

In Fig 10, we can see an upward trend in the number of active edges and nodes per week, confirming again the increasing activity of these farms.

**Fig 11:**
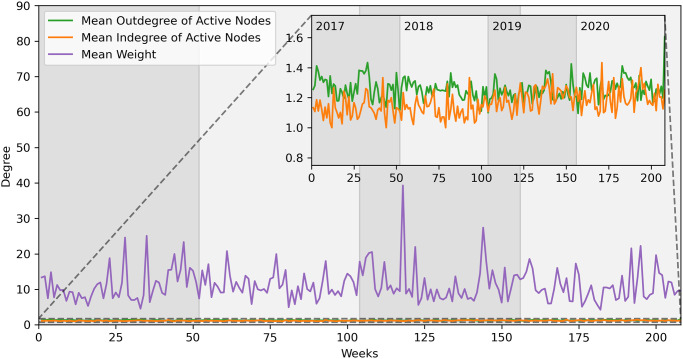
Buffalo network statistics—Weekly aggregation. Main plot: mean outdegree, indegree and weight. Secondary plot: magnification of outdegree and indegree.

In Fig 11, we observe the average outdegree, indegree and number of animals moved (weight). In the background plot we can see how the number of animals moved is bigger on average than the cattle network. In the foreground, we observe in a zoomed plot how the degrees have low and similar values, once again an indication of a network with low activity.

These nodes are less connected compared to those in the bovine network. Indeed, the largest weakly connected component, encompasses more than 95% of the nodes but the largest strongly connected component is composed by a bit more than 25% of all the nodes.

### Mixed farms in the buffalo network

**Fig 12:**
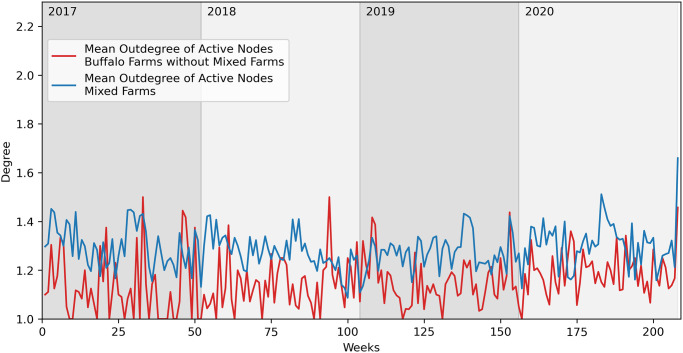
Comparison of mean outdegree of farms with only buffaloes and mixed farms.

**Fig 13:**
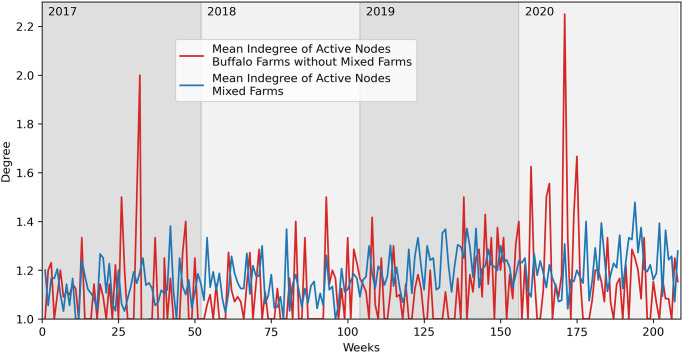
Comparison of mean indegree of farms with only buffaloes and mixed farms.

In Figs 12 and 13, we compare the buffalo network properties, respectively the mean outdegree and the mean indegree, of *mixed* farms to farms that raise only buffaloes. The average indegree of the two networks is similar, while the *mixed* farms have a higher average outdegree, an aspect that could, once again, impact the diffusion of a pathogen capable of reaching these farms.

Therefore, *mixed* farms are significant not only for providing a link to the cattle network but also for potentially accelerating the spread of a pathogen, given the above-mentioned considerations.

## Simulations and results

We simulated the diffusion of Q fever in our aggregated network, as seen in Fig 3. As depicted in Table 4, we considered various probabilities of infection and recovery. Starting the simulations we infected a different number of farms selected among different sets. For the latter, the percentage is relative to the total number of nodes in the aggregated 4-year network.

**Table 4:** Parameters of the simulations. Starting infected set.

Probability of infection λ ∈ {0.075, 0.1, 0.25, 0.5}
Probability of healing *μ* ∈ {0.01, 0.025, 0.05, 0.075, 0.1, 0.125, 0.15}
Infected farms ∈ {Buffalo farms, Buffalo farms and mixed farms}
Initial Percentage, among **all** farms, of infected farms ∈ {0.05% (78 nodes), 0.5% (780 nodes)}

In light of the considerations in the previous sections, to assess whether the buffalo network alone could indeed facilitate the diffusion of a pathogen, we initiated the simulation at week 0 by infecting a randomly selected subset of nodes, choosing from either buffalo farms or buffalo farms and *mixed* farms. The first approach avoids prematurely establishing a link with the cattle network and prevents overestimation of the diffusion due to the higher outdegree associated with the *mixed* buffalo nodes. Therefore, we anticipate the second method to result in a higher diffusion of Q fever in the network. Subsequently, we proceeded to simulate the diffusion of Q fever using real movement data aggregated by week from 2017 to 2020.

**Fig 14:**
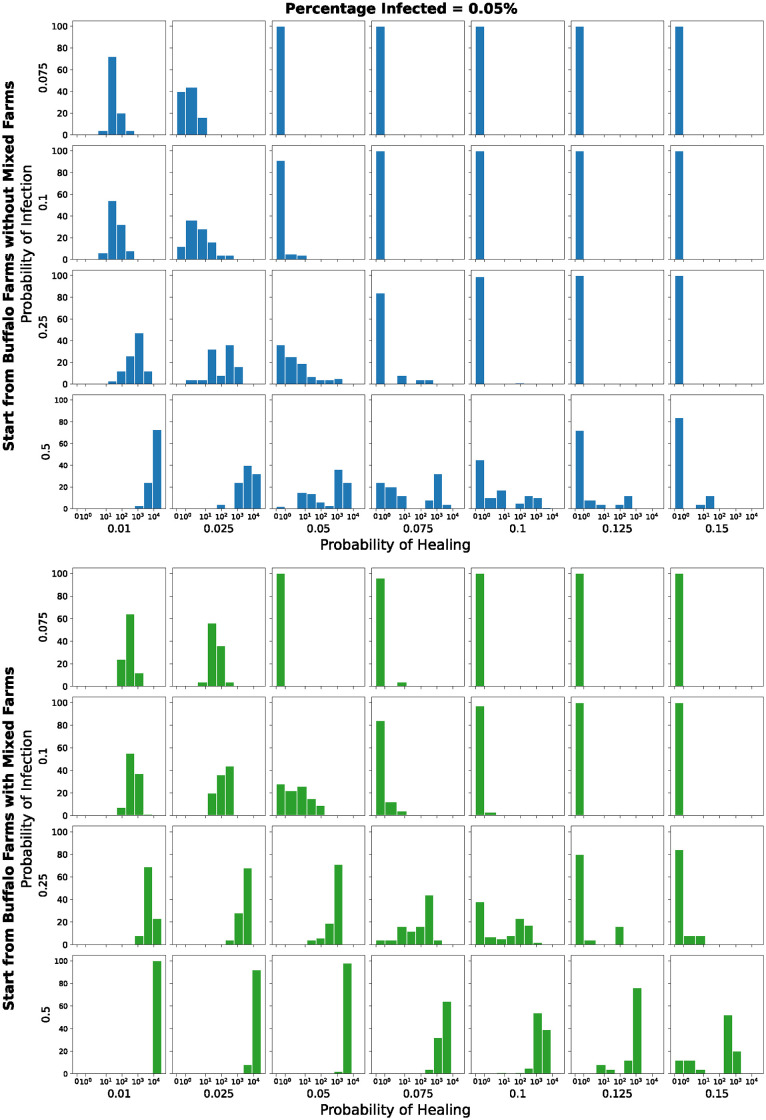
Number of infected farms, among 100 runs, at the end of each simulation. *Symlog* scale on the x-axis. Initial percentage of infected farms equal to 0.05%.

**Fig 15:**
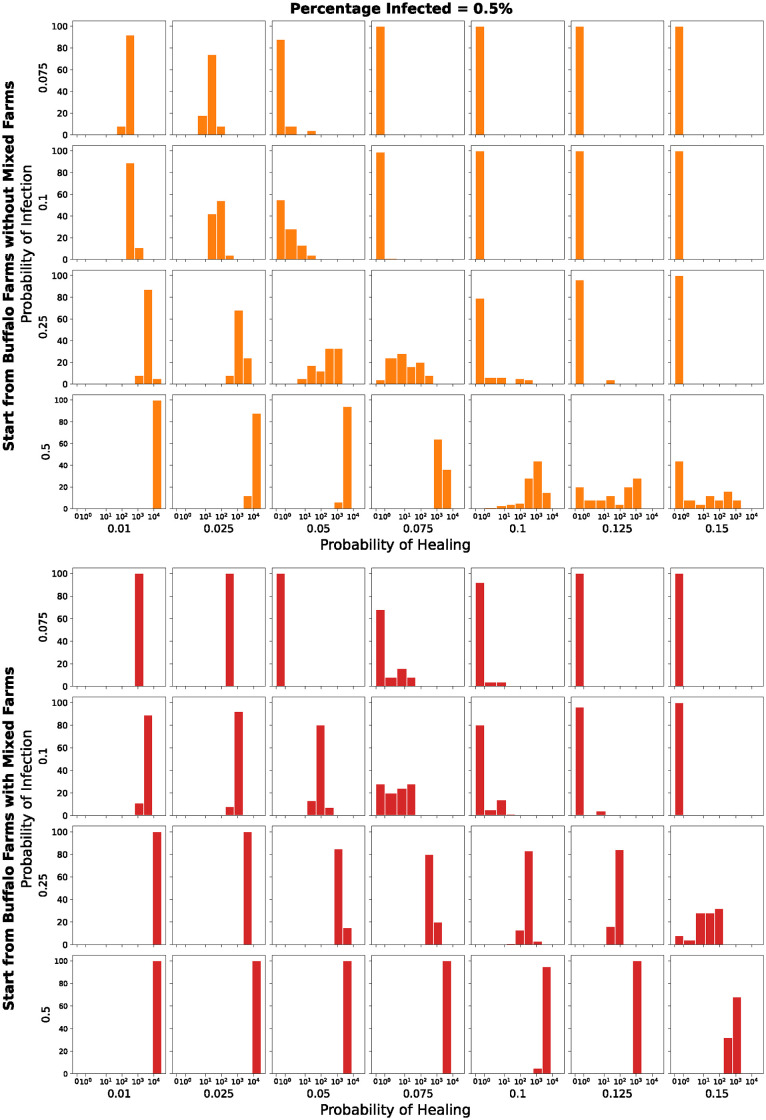
Number of infected farms, among 100 runs, at the end of each simulation. *Symlog* scale on the x-axis. Initial percentage of infected farms equal to 0.5%.

Figs 14 and 15 present 112 histograms, representing different scenarios of random selection, infection, and healing probabilities. The values described by the histograms indicate the number of infected farms at the end of each simulation among 100 runs and are shown using a *symlog* scale on the *x-axis*. They show the results of the simulations with the parameters in Table 4 and initial percentage of infected farms equal to 0.05% and 0.5% respectively.

The top grids (blue and orange) show the results of the simulations where the initial set of infected farms is composed of the buffalo farms without the *mixed* farms. The bottom grids (green and red), instead, show the results of the simulations where the initial set of infected farms is composed of the buffalo farms with the *mixed* farms.

In the top grid (blue) in Fig 14, we can see how only for a low probability of infection combined with a high probability of healing we do not have infected farms at the end of the simulation. In the other cases, we see how the histograms are located on values bigger than zero, denoting the presence, at the end of the 4 years, of farms that contain the pathogen. As expected, the number of infected farms increases when we increase the probability of infection. For example, we can see how in the three plots in the first column of the blue grid, the histograms shift to the right as we move down on the column. The opposite is true as we move right on a line.

In the bottom grid (green) in Fig 14 is interesting to see how starting from a set that contains also the *mixed* farms, so those farms that raise both buffaloes and cattle, allows the pathogen to spread from the buffalo network to the cattle network, therefore contributing to the spread to and among cattle farms. The general considerations are similar to the ones related to the top grid, but here, even when starting with a small percentage of infected farms, with a low probability of infection and a high probability of healing there are some runs where the pathogen is able to spread and survive into the cattle network.

In Fig 15, we can observe a similar behaviour to the one described just above. A higher initial number of infected farms results in fewer simulations with no infected farms at the end and a larger number of infected farms overall.

**Fig 16:**
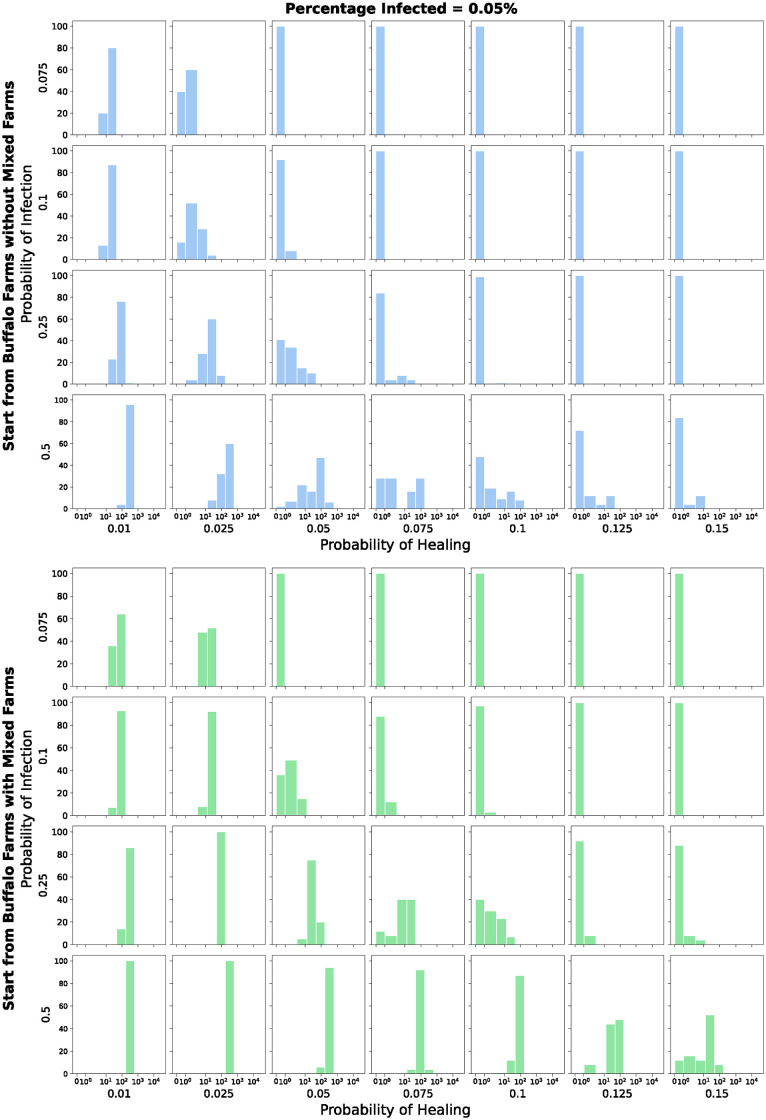
Number of infected *buffalo* farms, among 100 runs, at the end of each simulation. *Symlog* scale on the x-axis. Initial percentage of infected farms equal to 0.05%.

**Fig 17:**
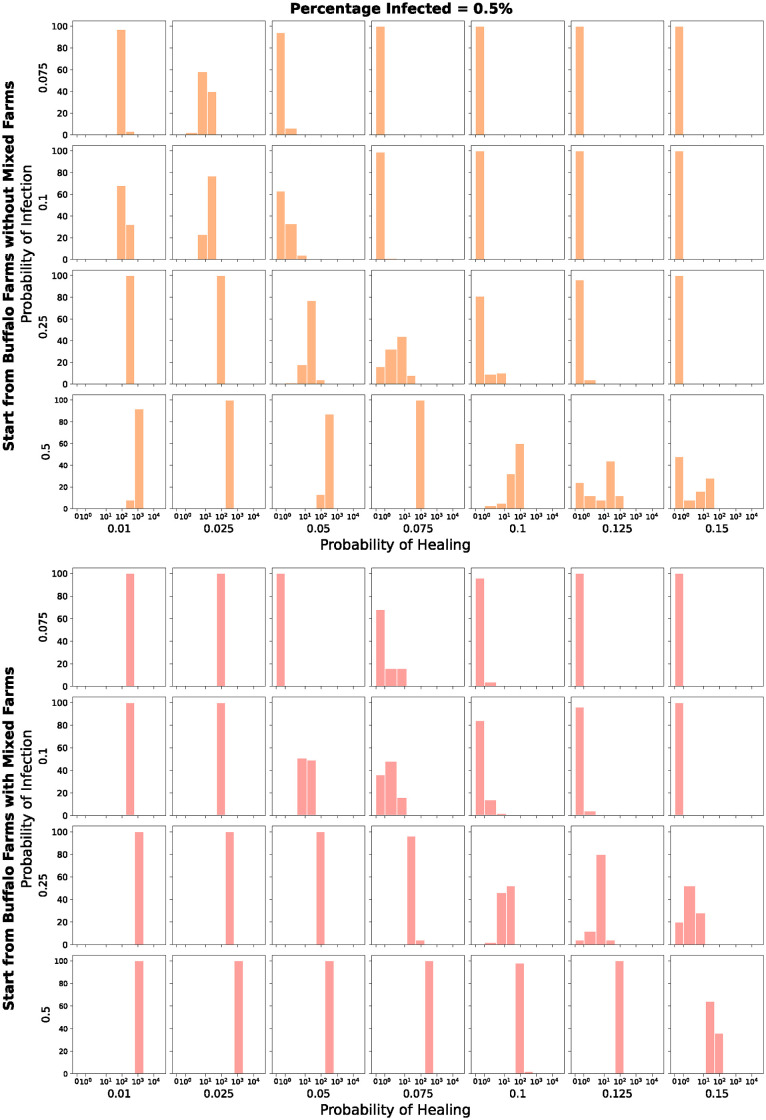
Number of infected *buffalo* farms, among 100 runs, at the end of each simulation. *Symlog* scale on the x-axis. Initial percentage of infected farms equal to 0.5%.

Figs 16 and 17 represent the same runs but the histograms describe the number of infected buffalo farms. The color scheme and the considerations are similar to the ones related to Figs 14 and 15 respectively, obviously with a different order of magnitude. But what is important to point out here is how also in a smaller network like the buffalo one, the pathogen is able to survive.

As anticipated, when the infections starts from both the buffalo farms and the *mixed* farms, the infection exhibits an easier diffusion into the cattle network. Nevertheless, the disease can still spread into the cattle network even with a small number of initially infected farms selected from the buffalo farms without the mixed farms. Furthermore, even with low infection probabilities, the pathogen, albeit less frequently, spreads into the cattle network, highlighting again a potential risk for this species.

**Fig 18:**
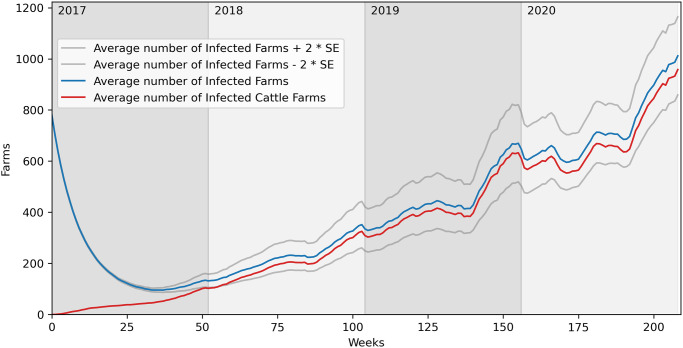
Average number of infected farms over 100 runs. λ=0.5, *μ*=0.1. Initial percentage of infected nodes equal to 0.5%. Nodes selected among the buffalo farms without the mixed farms. SE = Standard Error.

Fig 18 presents the average number of infected farms over 100 runs with parameters λ=0.5, *μ*=0.1 and starting by infecting 0.5% of all the nodes selected among the buffalo farms without the mixed farms (last row and fourth column in the orange histogram panel). Indeed, the blue line represents the number of infected farms. So at time 0, it will be composed of only buffalo farms. The total number of infected farms decreases at the beginning and then, around time 30, increases again. This is because as long as time goes on, while the number of buffalo infected farms decreases, an increasing number of cattle farms is infected, as we can see from the increasing behaviour of the red line. So, we can observe that the disease enters the cattle network and, from that point onward, it is able to spread.

**Fig 19:**
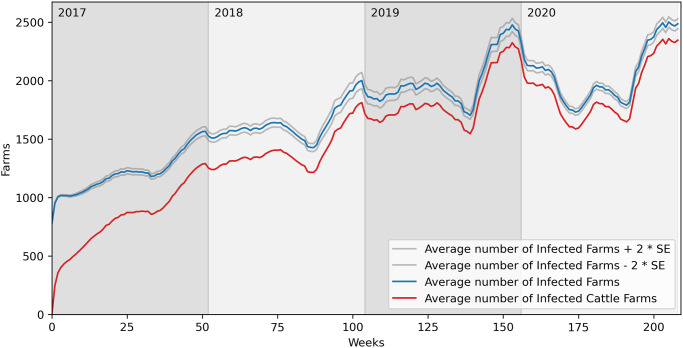
Average number of infected farms over 100 runs. λ=0.5, *μ*=0.1, Initial percentage of infected nodes equal to 0.5%. Nodes selected among the buffalo farms with the mixed farms. SE = Standard Error.

Fig 19 presents the average number of infected farms over 100 runs with the same parameters but the nodes are selected among the buffalo farms with the mixed farms (last row and fourth column in the red histogram panel). Therefore, at time 0, the blue line represents both buffalo and mixed farms. Unlike Fig 18, the blue line does not initially decline. Instead, there is an immediate increase in the number of infected cattle farms due to the presence of the pathogen in mixed farms, facilitating its spread within the cattle network.

To summarize, we have observed the capability of Q fever to spread within the cattle network, even when initiated from a small and poorly connected subset of farms that raise only buffaloes, so without infecting any cattle or *mixed* farms at the beginning. This holds even under a low probability of infection and a high probability of healing. It is also interesting to add how the pathogen survives also in the buffalo network.

## Discussion

While the buffalo network in Italy is considerably smaller than the cattle network, the close proximity of buffalo and cattle increases the risk of pathogen transmission.

Farms that contain both species effectively connect the two networks, facilitating the spread of the disease. Despite the substantial difference in the network sizes, the peculiarity of buffalo farms in Italy renders the buffalo network non-negligible when analysing the Italian bovine reality.

Indeed, we have seen how a pathogen can spread from the buffalo network to the cattle one. Excluding the buffalo network would therefore lead to a major loss of information and a consequent error in the analysis of the cattle network alone.

Given the recent case of Q fever in Italy [33] and its impact on humans, it could be useful to consider some kind of intervention on the *mixed* farms, either to obtain an early diagnosis of the Q fever or to reduce the risk of infection towards the bovine network that is often the cause of infection towards humans. The latter could be done by forcing the two species of animals into different spaces, reducing the contact between them, frequently cleaning the shared areas, and using different hays since they are a possible source of infection [45].

The hypothesis that buffalo farms in Italy act as a reservoir for the Q fever pathogen, *Coxiella burnetii*, is plausible due to several factors. Both cattle and buffalo farms share similar risk factors for Q fever spread. These include factors related to the animals themselves, such as age, stressful events, and pregnancies. While control measures for Q fever exist for both cattle and buffalo, the way they are implemented might differ. On average, buffalo farms tend to be smaller compared to cattle farms. This could potentially lead to less stringent biosecurity practices in buffalo farms, allowing for a higher prevalence of Q fever within those herds. Furthermore, the concentration of buffalo farms in southern Italy, where enforcing regulations can be more challenging, could contribute to a higher prevalence of Q fever. This situation is similar to what has been observed with other diseases like brucellosis and tuberculosis [46].

The presence of mixed farms, raising both cattle and buffalo, creates a potential transmission bridge between the species. If buffalo farms, due to the factors mentioned above, act as a reservoir with a higher prevalence of Q fever, these mixed farms could continuously reintroduce the pathogen back into the cattle network, hindering eradication efforts.

Buffalo, raised near cattle, may potentially contribute to the transmission of various diseases between the two species. Thus, the importance of buffaloes in Italy cannot be underestimated even in a broader scenario. Careful consideration is essential for understanding disease dynamics, not only for the specific pathogen under examination but also for different ones. In addition, the real case scenario of Q fever requires more attention since it can infect also other species.

Not only this, but also the fact that the pathogen can survive, as seen in most simulations, in the buffalo network, suggests that it is fundamental to consider both networks when trying to deal with Q fever.

So, as part of future work, exploring the interaction between buffalo, cattle, and other species like goats and ticks could provide a more comprehensive understanding of this phenomenon. These species could serve as reservoirs for pathogens, playing an important role. While not considered in this study, their inclusion in the model would contribute to a more accurate representation of disease spread, surely increasing its diffusion.

Moreover, the implementation of surveillance or prevention measures could be valuable in mitigating the risk of pathogen spread and safeguarding both animal and human health. Any potential control programs should consider all relevant species to effectively address the complexities of disease transmission.
